# Insurance status and traumatized patients’ outcomes: a report from the national trauma registry of Iran

**DOI:** 10.1186/s12913-023-09369-9

**Published:** 2023-04-24

**Authors:** Khatereh Isazadehfar, Payman Salamati, Mohammad Reza Zafarghandi, Vafa Rahimi-Movaghar, Moein Khormali, Vali Baigi

**Affiliations:** 1 Social Determinants of Health Research Center (SDH), Medical Faculty, Ardabil University of Medical Sciences, Ardabil, Iran; 2 Sina Trauma and Surgery Research Center, Tehran University of Medical Sciences, Tehran, Iran

**Keywords:** Insurance coverage, Trauma, Outcome, Admission, Length of stay

## Abstract

**Background:**

Trauma care is one of the most expensive medical procedures that is significantly affected by factors like insurance status. Providing medical care to injured patients has a significant impact on patients’ prognosis. This study examined whether insurance status was associated with different outcomes, including hospital length of stay (HLOS), mortality, and Intensive Care Unit (ICU) admission.

**Methods:**

This prospective study analyzed the data of traumatized patients who had been registered in the National Trauma Registry of Iran (NTRI), and hospitalized at Sina Hospital, Tehran, Iran, from March 22, 2016, to February 8, 2021. Given the type of insurance, the insured patients were classified as basic, road traffic, and foreign nationality. The outcomes of in-hospital death, ICU admission, and HLOS between insured and uninsured patients, and then different insurance statuses, were compared using regression models.

**Result:**

A total of 5014 patients were included in the study. 49% of patients (n = 2458) had road traffic insurance, 35.2% (n = 1766) basic insurance, 10.5% (n = 528) were uninsured, and 5.2% (n = 262) had foreign nationality insurance. The mean age of patients with basic, road traffic insurance, foreign nationality, and uninsured patients was 45.2 (SD = 22.3), 37.8 (SD = 15.8), 27.8 (SD = 13.3), and 32.4 (SD = 11.9) years, respectively. There was a statistically significant association between insurance status and mean age. Based on these results, the mean age of patients with basic insurance was higher than other groups (p < 0.001). Additionally, 85.6% of the patients were male, with male to female ratio of 9.64 in road traffic insurance, 2.99 in basic insurance, 14.4 in foreign nationality, and 16 in uninsured patients. There was no statistically significant difference between in-hospital mortality in insured and uninsured patients, 98 (2.3%) vs. 12 (2.3%), respectively. The odds of in-hospital mortality in uninsured patients were 1.04 times the odds of in-hospital death in insured patients [Crude OR: 1.04, 95%CI: 0.58 to 1.90]. Multiple logistic regression showed that after adjusting for age, sex, ISS, and Cause of trauma, the odds of in-hospital death in uninsured patients were 2.97 times the odds of in-hospital death in insured patients [adjusted OR: 2.97, 95%CI: 1.43 to 6.21].

**Conclusion:**

This study shows that having insurance can change the ICU admission, death, and HLOS in traumatized patients. The results of this study can provide essential data for national health policy for minimizing the disparities among different insurance statuses and proper use of medical resources.

## Background

Trauma is a major international health problem that causes more than 5 million deaths annually, roughly equivalent to deaths from certain infectious diseases such as tuberculosis, AIDS, and malaria [1]. More than 1.2 million fatal injuries happen on the world’s roads every year [2]. Although trauma is a significant cause of mortality and morbidity in developed and developing countries, more than 90% of injury deaths happen in low- and middle-income countries (LMICs) [1, 3]. Trauma injuries are among the top 10 causes of death worldwide [4]. Notably, some researchers predict that death from trauma will increase to the fourth leading cause of death by 2030 [5]. According to the GBD study for the year 2019, Iran had the highest mortality rate from road traffic accidents, and its death ranked second among all countries, and the percent change from 2009 to 2019 was 24.5% [6]. The cost of road traffic accidents in Iran is about 2% of GNP per year [7].

After cardiovascular diseases, trauma care is one of the most expensive medical procedures [8]. These measures include hospitalization, stay in the intensive care unit (ICU), and paraclinical and surgical procedures to diagnose and treat trauma patients. The Hospital Length of Stay (HLOS) is a qualitative factor in evaluating hospital care and physician performance [9]. Many clinical and nonclinical factors significantly influence HLOS [10]. However, injury severity score, comorbidities, and trauma mechanism are associated with HLOS and patient outcomes [11, 12].

Some previous studies have identified that injury severity and insurance-type factors act as predictors of length of staying for injuries [13, 14]. The relationship between HLOS and insurance status in trauma patients is equivocal. Some old studies have indicated no relationship or increased HLOS in uninsured patients [15]. However, some studies demonstrated that uninsured patients had significantly shorter hospital lengths of stay than insured patients [10, 16, 17]. According to the existing rules in Iran, all hospitalized injured patients due to traffic accidents are included in a particular type of insurance called road traffic insurance, in which all of their costs will be free of charge during the hospitalization. Moreover, all people of foreign nationality who reside in Iran can use specific health insurance called foreign nationality.

Hospital payment status is an essential factor in receiving health care. Consequently, the outcome of patients, such as mortality and even admission to the ICU, if needed, can be affected by the patient’s insurance status [18]. Previous studies have shown that the ICU stay in uninsured patients is shorter than in insured patients [19]. Some studies also emphasize the higher mortality rate of uninsured patients than insured patients [19, 20]. According to some studies, uninsured patients are less likely to receive an operation and have a poor quality of hospital care, leading to worse health outcomes and more mortality [20]. These findings require further investigation to characterize whether the insurance status can lead to differences in the quality of hospital care for trauma patients.

We hypothesized that insurance status would affect HLOS, admission to ICU, diagnostic and therapeutic procedures, and mortality in injured patients using the National Trauma Registry of Iran (NTRI) data.

## Methods

### Research design and participants

The present study is a cohort study that analyzed the data of the NTRI performed at Sina hospital in Tehran, from March 22, 2016, to February 8, 2021. NTRI is designed as a hospital-based multi-center registration center [21]. This center’s patient data is classified into ten groups based on 109 variables [22].

All trauma patients with one or more traumatic injuries admitted to Sina Hospital (according to the 10th edition of the International Classification of Diseases (ICD-10)) were included. The other inclusion criteria were: hospital stays longer than 24 h, death after injury, or transfers from ICUs of other hospitals.

### Data collection and procedure

The insured patients were grouped by their payment status into one of the following insurance groups: road traffic, basic, and foreign nationality.

The main dependent variables were the length of stay (HLOS) in the hospital and trauma outcome. The independent variables were the demographic characteristics of the participants (including sex, age, education, and marriage status), type and mechanism of injury, and insurance status. Also, Injury Severity Score (ISS), surgery, and diagnostic and therapeutic procedure were used as independent variables to indicate the patient’s medical condition Death and ICU admission were considered as other outcomes of trauma.

In the registry, three approaches were applied to collect data. Data related to age, sex, marital status, education, cause of injury, and transport mode were collected through interviews with the patient or the patient’s companion. GCS is extracted from the medical records. ISS, ICU admission, hospital length of stay, and patient outcome, including hospital death or discharge, discharge date, and insurance status, were also extracted from the hospital information system (HIS). Two dedicated nurses (registrar) performed this process. After filling out checklists by the registrars, they were uploaded to the web-based portal of NTRI. One trained supervisor (physician) evaluated data quality regarding integrity, consistency, and accuracy. [21]

The patients’ ICD-10 codes were converted to the AIS injury scale in the next step. Furthermore, the severity of the injury was calculated according to the ISS criteria. Each AIS code is a 7-digit number that the 6-digit identifier before the decimal number indicates the type of damage (Pre-dot code), and the right digit indicates the AIS severity score (Post-dot code). Values have a range of 3–75. Injuries to each part of the body were given a separate score of 1 (minor) to 6 (mortal). ISS was calculated using the three most severely injured areas, squaring each number for these values, and adding the results. Each area with mortal injury (six) received a score of 75 as the maximum value [23].

### Statistical analysis

Nominal and categorical variables were described as frequencies and percentages. Normally distributed variables were described by mean ± standard deviation. The non-normal distributed data were presented as the median and interquartile range (IQR). The association between insurance status and the categorical or nominal variables was assessed using the chi-square test. Also, ANOVA and Kruskal–Wallis test were used to compare quantitative variables between different insurance statuses. Post hoc analyses were conducted using Bonferroni’s test. Univariable logistic regression models were used to assess the association between the type of insurance and dichotomous outcomes (ICU admission and death). In the next steps, this association was adjusted for potential confounders using multiple logistic regression. Also, the association between the type of insurance and HLOS was assessed by univariable and multiple quantile regression models. In the multiple regression model, we used the likelihood ratio test for variable selection. P ≤ 0.05 was considered statistically significant. Statistical analyses were performed using the STATA software version 15.0 (Stata Corp, College Station, TX, USA).

## Results

The total number of included patients was 5014. The mean age ± SD of the participants was 39.3 ± 18.6 years (ranging from 2 to 90). The mean age of individuals in different insurance groups was statistically significant. According to these results, the mean age of patients with basic insurance (45.2 ± 22.3) was significantly higher than other study groups, and patients with foreign nationality (27.8 ± 13.3) were significantly younger than other groups. 86% of patients (n = 4293) were men, 55% (n = 2774) were married, and 51% (n = 2547 cases) of cases were due to traffic accidents. Of them, 49% (n = 2458) had road traffic insurance, 35.2% (n = 1766) basic insurance, 10.5% (n = 528) were uninsured, and 5.2% (n = 262) had foreign nationality insurance. 63% (n = 3143) of the patients were employed. Based on the insurance status, participants’ demographic characteristics were presented in Table 1. The mean years of education between payment groups were significantly different (p < 0.001).

**Table 1:** Baseline Characteristics of the patients according to the insurance type. N (%), (N = 5014)

	Road traffic (n = 2458)	Basic (n = 1766)	Foreign nationality (n = 262)	Uninsured (n = 528)	p-value	Pairwise comparison
**Age, mean ± SD, (year)**	37.8 ± 15.8	45.2 ± 22.3	27.8 ± 13.3	32.4 ± 11.9	< 0.001	B > R, B > U, B > F, R > U, R > F, U > F
**Sex**					< 0.001	
Male	**2227 (51.9)**	1324 (30.8)	245 (5.7)	497 (11.6)		
Female	231 (32.0)	**442 (61.3)**	17 (2.4)	31 (4.3)		
**Education, mean ± SD (year)**	8.9 ± 4.1	7.7 ± 4.8	2.9 ± 3.8	9.5 ± 3.6	< 0.001	R > B, R > F U > R, U > B, U > F, B > F
**Marital status**					< 0.001	
Married	1429 (51.5)	1012 (36.5)	108 (3.9)	225 (8.1)		
Single	916 (47.7)	587 (30.6)	141 (7.3)	275 (14.3)		
Divorced	113 (35.2)	167 (52.0)	13 (4.0)	28 (8.7)		
**Nationality**					< 0.001	
Iranian	2356 (50.8)	1760 (37.9)	0 (0.0)	521 (11.3)		
Non-Iranian	102 (27.6)	6 (1.6)	262 (68.9)	7 (1.9)		
**Job status**					< 0.001	
Employed	1730 (55.0)	841 (26.8)	202 (6.4)	370 (11.8)		
Housekeeper	144 (28.7)	**328 (65.5)**	15 (3.0)	14 (2.8)		
Retired	102 (43.0)	126 (53.2)	0 (0.0)	9 (3.8)		
student	144 (42.5)	115 (33.9)	14 (4.1)	**66 (19.5)**		
Unemployed	**150 (44.0)**	**146 (42.8)**	15 (4.4)	30 (8.8)		
Others	188 (41.5)	210 (46.4)		39 (8.6)		

R: Road traffic insurance, B: Basic insurance, F: Foreign nationality insurance, U: Uninsured

Table 2 shows the difference between the number of services provided in the hospital to uninsured patients and all the health insurance groups. The surgical intervention percentage of all trauma patients was 81% (n = 4061), similar between groups (p = 0.96). There were statistically significant differences between the groups based on performing diagnostic procedures, including radiography, ultrasonography, and electrocardiography (ECG). The percentage utilization of ECG (P < 0.001) and ultrasonography (P = 0.02), as well as the proportion of fixation (P < 0.001), were higher in insured patients than uninsured patients. However, performing radiography was more in uninsured patients than insured patients (P = 0.004) (Table 2).

**Table 2:** Diagnostic and therapeutic procedures taken by trauma patients according to insurance status (n = 5014)

Procedure	Insured (n = 4486)	Uninsured (n = 528)	Total (n = 5014)	p-value
**Radiography**	3383 (75.4)	428 (81.1)	3811 (76.0)	**0.004**
**Fixation**	1491 (33.2)	60 (11.4)	1551 (30.9)	**< 0.001**
**ECG**	401 (8.9)	19 (3.6)	420 (8.4)	**< 0.001**
**Surgery**	3633 (81.0)	428 (81.1)	4061 (81.0)	**0.96**
**MRI**	201 (4.9)	16 (3.0)	217 (4.3)	**0.12**
**Ultrasonography**	1205 (26.9)	117 (22.2)	1322 (26.4)	**0.02**
**Angiography**	1902 (42.4)	229 (43.4)	2131 (42.5)	**0.67**

Table 3 shows the difference between the injury ISS, GCS, and the cause of trauma in insured and uninsured patients. According to these results, the proportion of ISS ≥ 9 in insured patients was higher than in uninsured patients (16.9% vs. 7.0%, p < 0.001).

**Table 3:** cause of trauma, ISS and GCS of trauma patient by insurance status (n = 5014)

	Insured (n = 4486)	Uninsured (n = 528)	p-value
**ISS**			**< 0.001**
< 9	3729 (83.1)	491 (93.0)	
≥9	757 (16.9)	37 (7.0)	
**GCS**			**0.85**
13 to 15	4308 (98.1)	508 (98.4)	
9 to 12	33 (0.8)	3 (0.6)	
3 to 8	50 (1.1)	5 (1.0)	
*Missing*	95	12	
**Cause of trauma**			**< 0.001**
**Road traffic crashes**	2949 (55.6)	53 (10.0)	
Fall	1075 (24.0)	61 (11.6)	
Stab/cut	666 (14.8)	322 (66.0)	
Blunt	174 (3.9)	23 (10.0)	
Others	77 (1.7)	39 (7.4)	

Crude death rates were 2.3% and 2.3% for insured and uninsured patients, respectively (P = 0.89). The odds of in-hospital death in uninsured patients were 1.04 times the odds of in-hospital death in insured patients [Crude OR: 1.04, 95%CI: 0.58 to 1.90]. Multiple logistic regression showed that after adjusting for age, sex, ISS, and Cause of trauma, the odds of in-hospital death in uninsured patients were 2.97 times the odds of in-hospital death in insured patients [adjusted OR: 2.97, 95%CI: 1.43 to 6.21] (Table 4). Also, after adjusting for age, sex, ISS, and Cause of trauma, the odds of in-hospital death in foreign patients were 3.63 times the odds of in-hospital death in the road traffic insurance group [adjusted OR: 3.63, 95%CI: 1.49 to 8.84] (Table 4).

The percentage of ICU admission in the uninsured group was 10.8% and in the insured group was 16.2%. In other words, the odds of ICU admission in uninsured patients was 0.63 times that in insured patients [OR: 0.63, 95%CI: 0.47 to 0.84, p < 0.001). However, after adjusting for age, sex, ISS, and cause of trauma as potential confounders, no statistically significant difference was observed between the two groups [OR: 0.92, 95%CI: 0.66 to 1.28, p < 0.18) (Table 4).

**Table 4:** Clinical Outcomes of Trauma Patients by Insurance status and type of insurance (N = 5014)

		N (%)	Crude OR (95% CI)	Adjusted^a^ OR (95% CI)
Death	**Insurance status**			
	Insured	98 (2.3)	1	1
	Uninsured	12 (2.3)	1.04 (0.58 to 1.90)	**2.97 (1.43 to 6.21)** ^*^
	**Type of Insurance**			
	Road traffic	41 (1.7)	1	1
	Basic	49 (2.8)	**1.68 (1.11 to 2.56)**	0.68 (0.37 to 1.24)
	Foreign nationality	8 (3.1)	1.85 (0.86 to 4.00)	**3.63 (1.49 to 8.84)**
	Uninsured	12 (2.3)	1.37 (0.71 to 2.62)	**3.08 (1.43 to 6.65)**
ICU admission	**Insurance status**			
	Insured	775 (16.2)	1	1
	Uninsured	57 (10.8)	**0.63 (0.47 to 0.84)**	0.92 (0.66 to 1.28)
	**Type of Insurance**			
	Road traffic	395 (16.1)	1	1
	Basic	280 (15.9)	0.98 (0.83 to 1.16)	**0.63 (0.50 to 0.79)**
	Foreign nationality	50 (19.1)	1.23 (0.89 to 1.71)	1.46 (0.98 to 2.18)
	Uninsured	57 (10.8)	**0.63 (0.47 to 0.85)**	0.81 (0.58 to 1.14)

a: adjusted for Age, Sex, ISS, Cause of trauma

*Bold indicates p < 0.05

The median (IQR) HLOS was 127.5 (126.0) hours among the insured patients, while in uninsured patient’s median (IQR) HLOS was 92.5 (88.5) hours. Univariable quintile regression showed that the median of HLOS in uninsured patients was 35 h. less than the median of HLOS in insured patients (Estimate: -35, 95%CI: -43.9 to -26.1). Multiple quintile regression showed that adjusted for age, sex, ISS, and cause of trauma, the median of HLOS in uninsured patients was 22 h. less than the median of HLOS in insured patients (Estimate: -22, 95%CI: -31.1 to -12.9) (Table 5). Also, adjusted for age, sex, ISS, and cause of trauma, the median of HLOS in patients with basic insurance was 19.3 h. less than the median of HLOS in patients with road traffic insurance (Estimate: -19.3, 95%CI: -26.6 to -11.8).

**Table 5:** Hospital Length of Stay by Insurance status and type of insurance (N = 5014)

		Median (IQR)	Estimate (95% CI)	Adjusted^a^ estimate (95% CI)
HLOS	**Insurance status**			
	Insured	127.5 (126.0)	-	-
	Uninsured	92.5 (88.5)	**-35 (-43.9 to -26.1)** ^*^	**-22 (-31.1 to -12.9)**
	**Type of Insurance**			
	Road traffic	134.0 (125.2)	-	
	Basic	119.5 (122.0)	**-14 (-20.2 to -7.8)**	**-19.3 (-26.6 to -11.8)**
	Foreign nationality	115.5 (130.5)	**-18 (-30.9 to -5.1)**	-6.2 (-21.3 to 8.9)
	Uninsured	92.5 (88.5)	**-41 (-50.3 to -31.6)**	**-29.2 (-39.9 to -18.45)**

a: adjusted for Age, Sex, ISS, Cause of trauma

*Bold indicates p < 0.05

## Discussion

This study analyzed the factors associated with HLOS for trauma patients based on insurance status using NTRI data. The findings demonstrated significant differences in HLOS according to insurance status in trauma patients. Also, adjusted for age, sex, ISS, and Cause of trauma, the odds of in-hospital death in uninsured patients were higher than the odds of in-hospital death in insured patients. The median of HLOS in uninsured patients was 22 h. less than the median of HLOS in insured patients.

A recent study measured the length of hospital stay as a quality indicator of health care and proportionated costs. The average HLOS was reported for five days, which was almost in accord with others in the literature [24, 25]. However, the mean HLOS in some articles was lower than our result [26]. In contrast, previous studies showed that [10, 27] the average HLOS was longer than the current study. The discrepancy can be considered due to the different management, mechanisms, and the severity of the injury. Our study found that uninsured patients remained in hospitals shorter than insured patients. This result is consistent with evidence [16] that demonstrated that uninsured patients had significantly shorter HLOS than privately and publicly insured patients. Similar findings also were found in other studies done by Gardner R [13] and Conner KA [14]. We also found that uninsured patients remained in ICU for a significantly shorter length of time than insured patients. A study conducted among trauma patients in Arizona State showed that the overall and ICU HLOS in the hospital was significantly shorter in self–pay patients than in insured patients. [19]

The findings of our study were consistent with the previous studies [10, 16, 17] that showed that the average HLOS increases with increasing ISS severity. Although the increased length of hospital stay seems rational in terms of disease severity, this increase was also significantly different between different payment groups, according to the results of our data analysis. In contrast, previous study [27] showed that HLOS has no significant difference among patients with a severe (ISS ≥ 16) or more critical (ISS 41–75) injury. Therefore, severely injured patients receive sufficient treatment to alleviate their health problems, despite the type of health insurance.

Comparison in the present study in different insurance groups has a series of confounding factors. In other words, it is not just the payment method that affects mortality; there are other factors. Therefore, we made this comparison in different layers of trauma severity because the severity of the injury is one of the most important determinants of mortality. However, we used multiple regression models to adjust potential measured confounders. Due to the significant difference in the age distribution of patients in our data analysis and the higher average age of patients in insured patients, a longer length of stay in this group can be attributed to older patients and perhaps individual comorbidities [28]. Some studies have mentioned that different comorbidities such as pulmonary and neurologic diseases [29] and hypertension, peptic ulcer disease, and alcohol abuse [30] could influence the HLOS of trauma patients.

Another factor influencing the duration of hospital stay is undergoing therapeutic and diagnostic procedures for trauma patients, which can naturally extend the length of stay [26, 30]. The results of our study showed that the percentage of those who undergo surgery was not different among in payment groups. However, the procedures such as ECG, sonography, and fixation in insured patients were higher than in uninsured patients. Conversely, radiography was used more in uninsured than insured patients. This discrepancy can affect the outcome of uninsured patients because it may be challenging to diagnose injury in uninsured patients. Uninsured patients are less likely to receive appropriate health care due to the high costs associated with their treatments, as shown in previous studies [18, 31, 32]. Consequently, the health outcomes of these patients are worse, and they are more likely to suffer mortality than insured patients. [33, 34].

Insurance systems have various financial protection for diverse medical conditions. This protection provides more healthcare resources such as longer HLOS, paraclinical procedures, and other inpatient care for insured patients [35]. The lower services coverage could elevate out-of-pocket charges in uninsured patients, influencing their decision for treatment choices and treatment time, finally affecting the patient’s prognosis [36]. In our findings, although the mortality in uninsured patients compared to insured patients was more significant, even after controlling for ISS, this difference was not statistically significant. However, we must keep in mind that due to limited data, it cannot be concluded that insured people have better health outcomes or have access to better quality trauma-related care.

Our results suggested that the basic insurance group has significantly higher in-hospital mortality than patients with road traffic insurance. The reason may be due to the higher mean age of this group of patients compared to the other two groups. One possible explanation for these disparities in health outcomes across health insurance groups may be differences in government funding in various insurance systems [18]. We also found that the road traffic insurance group had longer HLOS than the two other groups; this was probably due to a large number of patients with more severe trauma (ISS ≥ 9) in this group of patients.

The strength of this study is using the NTRI as a representative, accurate significant data source of trauma patients, which analyzes data at different time intervals and has been refined by experts. There are several limitations to this study. First, we could not be able to account for pre-existing comorbidities of the patients. Second, the NTRI was a hospital-based project, which might not be a comprehensive representative for trauma patients in the general population. Finally, there might be uninsured patients or even patients with basic insurance covered by this insurance in the road traffic group because they had an accident. Therefore, these limitations should be noticed when interpreting and popularizing the results of this study.

Nonetheless, our results draw attention to disparities in care for traumatic injuries among uninsured patients compared to insured ones. Therefore, this study underlines the importance of systematically recording information on all comorbid conditions in trauma registries. Future research studies should investigate the effect of comorbidity on different trauma outcomes. Also, future research is necessary to adjust HLOS for clinical and nonclinical factors for using it as a valid outcome measure. In future studies, focusing on pre and post-hospital information is warranted.

## Conclusion

This study indicates that insurance status strongly affects post-trauma HLOS, even after adjusting for the effects of known covariates such as ISS, suggesting that health care costs impact medical care utilization. Uninsured patients were discharged earlier and had poorer health outcomes consistent with previous literature. Therefore, it is necessary to consider appropriate strategies and interventions for patients’ management during hospitalization.

## Data Availability

Data supporting the findings of this study are not publicly available. However, data are available from the corresponding author upon reasonable request (contact through the corresponding author).

## List of Abbreviations

NTRI: National Trauma Registry of Iran
LMICs: low- and middle-income countries
ICU: Intensive care unit
HLOS: Hospital Length of Stay
HIS: Hospital Information System
ICD: International Classification of Diseases
ISS: Injury Severity Score
AIS: Abbreviated Injury Scale
